# Does the bankrupt cheat? Impact of accounting manipulations on the effectiveness of a bankruptcy prediction

**DOI:** 10.1371/journal.pone.0280384

**Published:** 2023-01-17

**Authors:** Przemysław Mućko, Adam Adamczyk

**Affiliations:** Institute of Economics and Finance, University of Szczecin, Szczecin, Poland; University of Almeria: Universidad de Almeria, SPAIN

## Abstract

The aim of this article is to answer the question whether the unreliability of the Altman bankruptcy prediction model may be caused by manipulations in financial statements. Our study was carried out on a group of 369 bankrupt Polish companies, with the research period covering the years 2011–2020. In the study, we divided the companies into two groups: those correctly classified by Altman’s model as at risk of bankruptcy, and companies for which the model did not indicate a significant bankruptcy risk. Using a logit model, we tested whether the probability of companies being correctly classified as failed depends on the risk of a manipulation of financial statements. We use Benford’s law to measure the risk of a manipulation of financial statements. We also repeated our study using panel data models. Our analyses show that the manipulation of financial statements is not the cause of the inaccurate predictions of the Altman model. On the contrary, the results of the analyses indicate that manipulations occurs for companies with a lower Z-score and therefore a worse financial situation. This means that a deterioration in the quality of financial statements can be a signal of an increasing probability of bankruptcy.

## Introduction

Bankruptcy prediction models are valuable analytical instruments used by entrepreneurs, financial institutions, and individual investors alike. However, despite the use of increasingly sophisticated methods, their effectiveness still needs to be improved.

There may be many reasons for the unreliability of bankruptcy models. Some bankruptcies cannot be predicted because they are caused by sudden, unusual, and unexpected events. In some cases, the problem may be caused by poorly selected or applied bankruptcy prediction methods [1]. There is also the possibility that errors in bankruptcy predictions are related to the poor quality of data published in financial statements. Unreliable data can result from both errors made by accountants unintentionally and deliberate manipulations of financial statements.

The bankruptcy prediction models used so far have been based on the tacit assumption that financial statements reflect the financial condition of companies well [2]. However, such an assumption is not necessarily correct. There are premises to expect that companies in financial distress may intentionally manipulate financial statements [3–5]. Such action may be related to attempts to avoid indirect costs of bankruptcy (such as loss of customers, loss of employees), as well as the possibility of externalizing the costs of bankruptcy into the environment due to the phenomenon of bankruptcy fraud [6]. Investigating the relevance of a financial statement manipulation to the validity of bankruptcy prediction models is hampered by the problem of clearly determining whether financial statements have actually been manipulated [7, 8].

The most recent attempts to integrate financial statements manipulation proxies into bankruptcy prediction models are based on the concept of earnings quality [6, 9]. Since earnings quality measures have serious drawbacks in our research context, and do not cover the entire problem of financial statements manipulations [8, 10], we applied measures that are based on Benford’s law [10, 11].

The purpose of this article is to determine whether manipulation of financial statements can be a significant cause of the unreliability of bankruptcy prediction models. To answer this question, we used a sample of 369 Polish companies. The study period is 2011–2020. For the adopted sample, we analysed the factors affecting the Altman model’s correct classification of companies as failing. Among these factors, we include measures of the consistency of the distribution of the first significant number of financial data with the Benford distribution. These measures allow us to determine the probability of the financial statements being manipulated. We expect that the probability of correctly classifying a company as bankrupt should be negatively correlated to measures of the conformity between the actual distribution of significant figures and the Benford distribution. In other words, we hypothesize that misclassified companies have manipulated financial statements. To verify this hypothesis, we used logit models and panel data models.

The originality of our article lies in combining bankruptcy prediction models with methods to detect accounting manipulations, based on Benford’s law. Our main findings justify the inclusion of additional factors in the bankruptcy prediction models of accounting manipulation, i.e. proxies that has a broader scope than earnings quality, which were proposed in other research [6, 9]. We applied the measure that encompasses both intentional manipulations and unintentional errors, built on the basis of Benford’s law.

## Literature review

### Impact of data quality on the effectiveness of bankruptcy prediction models

Although the problem of a bankruptcy prediction was first presented in financial literature more than 50 years ago, it continues to receive almost unflagging interest to this day [1, 12, 13]. Since the publication of the first works by Beaver [14] and Altman [15] there has been a discussion about the most effective methods of bankruptcy prediction [1], the need to profile them to take into account the specifics of the industry and the country in which companies operate [16]. The need to constantly update models as they adapt to new business conditions is also being considered [17].

Due to the rapid technological development in recent years, many works have appeared, proposing h new techniques to be used, such as logistic regression, discriminant analysis, k-nearest neighbour, neural networks, decision trees, support vector machines, and least-squares support vector machines [1, 18]. However, despite the use of increasingly sophisticated methods, the accuracy of indications of bankruptcy prediction models has not been significantly improved, and the longest used Altman model is still considered a very effective tool for identifying companies at risk of bankruptcy. Altman et al. [16] demonstrated the high versatility of the model by claiming that the general Z-score model works reasonably well for most countries (the prediction accuracy is approximately 0.75) and the classification accuracy can be improved further (above 0.90) using country-specific estimation that incorporates additional variables [16].

The lack of significant improvement in the discriminatory characteristics of bankruptcy prediction models, despite the use of increasingly sophisticated methods, may mean that the cause of misclassification of companies is not due to the weakness of the methods applied, but to the quality of the data used for this purpose. The research results indicate that it is possible to increase the detection rate for bankrupt companies by eliminating the outliers reported for companies that continue to operate as a going concern [19]. Similarly, Tsai and Cheng [20] demonstrate that removing outliers improves the predictive accuracy of models. According to Wang and Liu [21] it is feasible to improve the model performance by increasing the undersampling rate. On the other hand, Karpoff et al. [22] underlie the drawbacks of data bases used in bankruptcy modelling: data omission, especially just before the year of bankruptcy and especially in the case of smaller or younger companies. Thus, they emphasize that the quality of data is crucial for the purpose of bankruptcy prediction.

The problem of the quality of financial data arises in many accounting studies. Generally, researchers tend to assume that financial data reflect a true and fair view of the financial situation and performance of reporting entities. This assumption holds even in research when the main phenomena may be closely related to errors or manipulations in financial statements, as in the case of the prediction of bankruptcy [3]. Serrano-Cinca et al. [2] claim that the accuracy of bankruptcy prediction may be affected by deliberate actions of managers that distort the picture of the financial situation. In their opinion, the majority of studies that try to predict business failure assume that accounts give a true and fair view of the financial position of a company, without considering that managers can discretionarily apply accounting rules or even perform accounting fraud. According to their study, several indicators proposed in the literature as earnings management proxies showed statistically significant differences between failed and non-failed firms [2]. Therefore, it is natural to assume that financial distress is a significant incentive to manipulate financial statements [5].

Financial distress is considered as one of the factors that motivate managers to distort financial statements [7]. Thus, recent research focuses particularly on financial reporting quality as an additional variable missing in bankruptcy prediction models. Financial reporting quality is a complex concept that was measured with earnings quality or accruals quality proxies in bankruptcy research. For example, Ashraf et al. [6] constructed a distress prediction model for companies from the UK and Pakistan. In their research, both earnings quality and accrual quality measures are positively correlated with the bankruptcy probability, which means that higher level of earnings management is typical for companies in distress. Costa el al. [9] presented a financial default prediction model for small and medium private companies from Portuguese construction industry. They showed that the inclusion of financial reporting quality proxy improves the models. However, of their three proxies of financial reporting quality, only accruals quality and earnings smoothness are significant (whereas earnings timeliness is not). Moreover, the coefficient of the accruals quality variable is negative, suggesting that the more discretionary accruals, the lower the probability of bankruptcy.

Both studies are relevant to our research, since both use data from less developed countries and both point to the quality of financial data as a necessary variable in the prediction of bankruptcy. However, accounting conservatism, which is also considered a measure of financial reporting quality, reduces the risk of bankruptcy [23]. Thus, we may conclude that the evidence is mixed on the sign but not on the significance of the influence of earnings quality on the financial statements manipulations. However, it is noteworthy that financial statements in these studies was measured with earnings quality proxy, which captures much, but not all, of the financial data quality phenomenon.

In many studies, both phenomena are analysed as one: The authors declare predicting bankruptcy or financial distress, although financial data quality is also captured within the model. Eventually, the successful prediction of bankruptcy is the aim of the phenomena. This is particularly true in case of the studies that rely solely on financial ratios, since both bankruptcy prediction and manipulation detection models use financial ratios extensively, if they use any other variables at all. Therefore, bankruptcy prediction models based on financial ratios can be improved when other variables are included [3]. Since bankruptcy and accounting manipulations can co-occur, to improve a model based on financial ratios (such as Altman’s models), another proxy for accounting manipulation, other than a financial ratio, is necessary.

### Assessment of financial reporting manipulations based on Benford’s Law

Manipulations in financial statements represent one of the dimensions of the quality of financial statements [24]. Generally, high-quality financial statements do not contain a significant portion of purposeful errors. However, the earnings quality models have quite significant drawbacks that make it difficult to use them to correct bankruptcy prediction models for the purposes of this article. They are not adequate for the purposes of our research since they rely on financial ratios (e.g. bankruptcy prediction models). Thus, potential errors and misstatements may be captured within the bankruptcy model. Moreover, they result in measures that correlate with underlying firm characteristics and rely on time-series, cross-sectional, or forward-looking data [10]. Furthermore, these methods are characterized by high information requirements based on data not available for the analysis of financial statements of smaller entities [3].

An alternative to classic fraud detection models, devoid of most of their disadvantages, is a method that exploits natural regularities occurring in data sets, called Benford’s law. Benford’s law refers to the observation that in many collections of numbers, the leading significant digits are not uniformly distributed but follow a particular logarithmic distribution [25]. It was initially observed over a hundred years ago by Simon Newcomb and later independently by Frank Benford, after whom it has been named [26, 27]. Benford tested it on various data sets, such as street addresses, numbers appearing in a magazine, and river drainage areas [11, 28]. All of them follow the same pattern, i.e. the probability that the first digit is 1 was about 30.1%, while the probability of 9 was 4.6%. According to Benford’s law, the probability that a digit *d* is the first digit of a number is equal to:

$$P(D=d)=Log_{10}(1+d^{-1})$$ (1)

where: *d* ∈ {1,2,…,9}.

Benford’s law has been tested on many data sets. For example, outside economics and management, Benford’s law was applied to test, inter alia, frauds in elections [29], quality of geological data [30], or social and behavioral characteristics of social media [31]. In economics and finance, Corazza, Ellero, and Zorzi [32] showed the conformity of the prices and returns of S&P 500 stocks; the rare non-conformity was associated with events such as stock exchange crashes. Skousen, Guan, and Wetzel [33] revealed the conformity of the annual earnings of Japanese companies from 1974 to 1997. Benford’s law was also used to verify LIBOR manipulations [34, 35]. In auditing, Nigrini and Mittermaier [28] showed the usefulness of Benford’s law in the analytical procedure for external and internal audit, which was later repeated or approved in many other studies [36, 37]. Benford’s law was also applied to reveal earnings management practices in New Zealand [38], the US [39], and Finland [40]. Although it is widely used in many fields and has been shown to fit many datasets, Benford’s Law is not supported by rigorous formal mathematical proof [41]; only some steps toward its fundamental understanding were achieved [41, 42].

However, for the purposes of the article, it is sufficient that the following is proved: If distributions are selected at random and random samples are then taken from each of these distributions, the significant digits of the combined sample will converge to the logarithmic (Benford) distribution [43]. Therefore, the non-conformity of a financial data set with Benford’s law should raise some level of suspicion [44]. Intentional distortion of the initially Benford-compliant data set moves it further from Benford distribution. If manipulation aims to increase actual amounts, then the percentage of first large digits in manipulated data will be greater than expected according to Benford’s law, and the percentage of first small digits observed will be less than according to Benford’s law. The reverse would be the case when reducing numbers: we would expect that smaller numbers are favoured and larger numbers to be underrepresented [45]. Therefore, measuring conformity with Benford’s law may be useful when assessing the extent of data manipulation. In the Methods section, two standard measures of distribution conformity are explained and applied: χ^2^ and MAD.

## Hypothesis

Our main research question is whether accounting manipulations and errors are a significant factor that causes Altman’s model to fail. Altman’s Z-score may be interpreted as a measure of the financial situation. Since, earnings management tends to occur more intensively in companies in distress, with worse financial performance, and more indebted [24], Altman`s Z-score is negatively correlated with the probability of accounting manipulations. However, we expect that, in the case of future bankrupt companies, a significant number of them are able to manipulate the financial statement to such an extent that the Altman model is incapable of predicting their bankruptcy.

In other words, based on the above literature review, we formulate a hypothesis approaching the problem of the unreliability of the Altman model. According to our hypothesis:

*H1*: *The Altman model prediction error occurs due to the manipulation of accounting data presented in the companies*’ *financial statements*.

Management of companies in a bad financial situation and potentially heading for bankruptcy are under pressure to improve the financial performance or debt of the entity and, therefore, are more likely to provide manipulated financial statements.

To answer the question whether financial statement manipulation may have a significant impact on the effectiveness of bankruptcy prediction, we combined bankruptcy prediction models with a methodology for assessing the likelihood of accounting manipulation.

## Methods

The first stage of our study was to check whether the bankruptcy of the selected companies was correctly predicted by the Altman model. Of the three existing variants of the Altman model, we adopted the four-factor model for emerging markets from 1995, expressed by the formula [46]:

$$Z"=3.25+6.56X_{1}+3.26X_{2}+6.72X_{3}+1.05X_{4}$$ (2)

where

X_1_-Working Capital/Total Assets;

X_2_-Retained Earnings/Total Assets;

X_3_-Earnings before Interest and Taxes/Total Assets;

X_4_- Book Value Equity / Total Liabilities

The advantage of this variant over previous versions of the model stems from the use of only financial statement data instead of market valuation data, which makes it applicable not only to publicly listed companies (as was the case in the first variant of the Altman model), but also to unlisted companies. Since most bankruptcies involve small non-public entities, this is another advantage of the variant. Furthermore, the four-factor model eliminates the fifth component (asset turnover ratio), the value of which is largely sector-specific [16]. As a result, the model can be applied to companies in a variety of industries.

The aim of the study was to answer the question whether the Altman model’s incorrect indications of failure to detect the risk of bankruptcy can be caused by manipulations/errors in the financial statements. To verify the hypothesis in the question, a binary variable was used, the value of which depended on whether the bankruptcy prediction model correctly identified the risk of bankruptcy in a given case. The assignment of individual cases to the group of correctly and incorrectly identified cases caused difficulties. This was due to the fact that, in the case of the four-factor Altman model used in the study, no specific Z-score threshold values are given to clearly identify entities at risk of bankruptcy. A cut-off value of 0 was used as the average value for companies with a default equivalent rating [46]. If the model correctly classified the company as *bankrupt*, the binary variable was assigned a value of 1 otherwise 0 (*Dummy* variable *Z2*).

We then built a logit model that enables us to identify the factors that influence the probability of correctly classifying companies as bankrupt. In this model, the key explanatory variable is a measure to determine the probability of a manipulation of the financial statements. If the manipulation is the cause of incorrect indications in the bankruptcy prediction model, this variable should be negatively correlated with the binary variable that determines the correctness of the classification of companies as bankrupt.

An important problem was the choice of method to detect accounting manipulation. We applied measures of the conformity of the distribution of significant digits of the values reported in the company’s financial statements with the Benford distribution as the main explanatory variable. Following the solution developed by Amiram, Boznic and Rouen [10], we calculated measures of conformity to the Benford distribution for the numbers found in each set of financial statements in the research sample. As the literature points to at least several possible measures of conformity of empirical distributions with the Benford distribution for the study the two most commonly used measures were applied. One of them is the χ^2^ determined according to the formula.

$$\chi^{2}=n\overset{9}{\underset{d=1}{\sum }}\frac{{\left(W_{d}-p_{d}\right)}^{2}}{p_{d}}$$

Where

n–sample (data set) size

w_d_−relative frequency of the number d as the first non-zero digit in a numerical set of n elements

p_d_−probability of a digit d occurring in the first significant position in a Benford distribution.

We used the χ^2^ measure to assess the distance of the empirical distribution from the Benford distribution; we did not test the hypothesis of their conformity. However, a frequently cited disadvantage of this measure is its high rigorousness: With a large sample size, it is too sensitive to the presence of small deviations from the Benford distribution [26, 47, 48]. Therefore, it is postulated that another measure should be used that does not depend on the size of the data set. Therefore, the MAD measure (*Mean Absolute Deviation)* proposed by Nigrini [11], which has been used in many other studies [10, 45, 47, 49–51], was also used. The MAD is determined as the average of the absolute differences between the expected and empirical digit frequencies. In studies on the first digit, the MAD is calculated as follows.

$$MAD=\frac{1}{9}\overset{9}{\underset{d=1}{\sum }}\left|w_{d}-p_{d}\right|$$

where

Wd–observed in the n-element set relative frequency of the digit d in the first significant position,

pd–probability of d to occur as the first non-zero digit in a number according to Benford’s law.

The higher the value of both of the above measures, the lower the conformity of the financial statement items with the Benford distribution, and, therefore, the higher the probability of financial statements manipulations.

The number of years to bankruptcy (YTB) was used as the first control variable. We expect that the coefficient of this variable should assume a positive value, which means that the ability of the Altman model to correctly indicate the probability of bankruptcy should increase as the moment of bankruptcy approaches. As a next variable, we took the number of items (N) from the financial statements considered to determine the MAD and χ^2^ values. The number of items in the financial statements analysed can affect the values of the χ^2^ statistic. Moreover, the number of non-empty items can also be considered as a simple measure of the quality of the financial statements: the more data in the report, the greater the amount of information, the higher the quality of the information, albeit in its very simple meaning. Similarly, at one stage in the development of research on the quality of non-financial information, the quantity of information (the size of non-financial disclosures, measured in terms of the number of characters or words) was used as a proxy for the quality of those disclosures [52]. Excessive textual information is also correlated with the likelihood of fraud [53]. Furthermore, the quality of the financial statements can depend on the size of the company, so the study also included a variable controlling for the size of the company expressed in terms of asset value (TA).

## Data

The study was carried out on a sample containing only bankrupt companies. Data for the study were taken from the Orbis database. We applied the legal definition (or ex-post criterion) of bankruptcy in the study [9, 54]. The year of bankruptcy (variable YTB = 0) is the year in which the company was marked as bankrupt in the Orbis database. The research period covered the years 2011–2020. In this period, there were 1207 Polish companies marked as bankrupt in the Orbis database, for which there was at least one financial statement. After balancing the data, 369 Polish bankrupt enterprises were obtained with data for four years before bankruptcy (plus one year when bankruptcy occurred). A total of 1845 observations were included in the analysis. In Table 1, we show the descriptive characteristics of the variables used in our models.

**Table 1:** Data characteristics.

	N	Mean	SD	Min	Q1	Median	Q3	Max
**Dummy Z2**	1845	0,65	0,48	0	0	1	1	1
**Z2**	1845	-12.78	25.03	-140.31	-16.49	-3.79	2.23	7.22
**MAD**	1845	0.06	0.02	0.01	0.05	0.06	0.07	0.14
**χ** ^**2**^	1845	13.62	7.65	1.14	8.42	11.96	16.81	66.59
**N**	1845	28.98	3.97	15	27	30	32	38
**TA** [million PLN]	1845	5.62	15.18	0.01	0.48	1.37	4.34	218.82
**YTB**	1845	-2	1.41	-4	-3	-2	-1	0

## Results

### Logit regression

Logit regression was performed on four models: including either MAD or χ^2^ as an explanatory variable reflecting (proxy) financial statement quality, and including or not including the time variable as a simple explanatory variable. Results are presented in Table 2.

**Table 2:** Logit regression results.

	Models			
	1	2	3	4
**χ** ^**2**^			0.0209687*** (0.0077154)	0.0165244** (0.0078682)
**MAD**	10.39306*** (3.615137)	8.253213** (3.772329)		
**N**	-0.1736938*** (0.0172409)	-0.1226561*** (0.0181608)	-0.1841904*** (0.0161748)	-0.1308927*** (0.0170438)
**TA**	0.0040282 (0.0033291)	0.0042297 (0.0034378)	0.0039412 (0.003327)	0.0041882 (0.0034416)
**YTB**		0.5141362*** (0.0423755)		0.514508*** (0.0423562)
**Const**	5.113906*** (0.6181971)	4.854827*** (0.6446206)	5.732756*** (0.5098033)	5.345092*** (0.5280192)
**Number of observations**	1845	1845	1845	1845
**Pseudo R2**	0.0813	0.1484	0.0811	0.1483

*Statistically significant at the 10% level

**Statistically significant at the 5% level

***Statistically significant at the 1% level

The estimated model shows that a high MAD and χ^2^ value favours the correct classification of companies as bankrupt (i.e. Z-score values below 0). Thus, the result is that the manipulation of financial statements is not the cause of the unreliability of the Altman model in correctly identifying bankrupts. Rather, there is a reverse effect: companies characterised by a weak financial position, and therefore a low Z-score, are at the same time characterised by poorer quality financial statements. Our analysis shows that the correct classification into the group of failed companies is not influenced by the size of the company. In contrast, the number of disclosures available in financial statements negatively affects the probability of detecting potential bankruptcy. The lower the quality of the financial statements, measured simply as the number of items disclosed, the worse the financial health of the company, as shown in the financial statements, and therefore the higher the probability of success of the Altman model.

### Pooled regression

Taking into account the fact that the results of a study based on a logit model may depend on the critical point adopted (the value that separates companies considered at risk of bankruptcy from those not at risk), an alternative analysis was also carried out, in which the Z-score was used as the explanatory variable. In this case, we used a panel data model with fixed effects. The type of model was chosen on the basis of the Hausman test. Results are presented in Table 3.

**Table 3:** Pooled regression results (fixed effects).

	Models	
	1	2
**MAD**	-76.24397** (31.62971)	-
**χ^2^**	-	-0.1072408* (0.0630644)
**N**	2.993145*** (0.1885966)	3.1144*** (0.1768628)
**TA (total assets)**	0.36508*** (0.0712026)	0.3617224*** (0.0712987)
**Cons**	-97.14783*** (6.37134)	-103.591*** (5.31544)
**R^2^ within**	0.2209	0.2194
**R^2^ between**	0.0614	0.0602
**R^2^ overall**	0.1064	0.1047
**Number of obs**	1845	1845
**Number of groups**	369	369

*Statistically significant at the 10% level

**Statistically significant at the 5% level

***Statistically significant at the 1% level

The results confirm the conclusions regarding the relationship between the financial situation of companies and manipulations in financial statements which was derived from the logit model: the value of the explanatory variable is negatively related to measures of manipulation. This means that for companies that went bankrupt while having a high Z-score, the measures of conformity to the Benford distribution had lower values, implying a low probability of fraud. At the same time, the relationship between the Z-score and the number of available financial statement items is positive, which may mean that greater detail in the financial statements is correlated with a better financial position of the company. Taken together, the results of both models and both variables representing the quality of the financial statements lead to the conclusion that the unreliability of the Altman model is due to reasons other than data quality.

## Discussion

We showed that data quality is a significant variable in bankruptcy prediction, although we could not verify our hypothesis positively. On the contrary, the sign of the estimated coefficients in both models means that the better financial situation of failed companies is negatively correlated with financial statements manipulations and errors. Thus, even in the face of the possibility of bankruptcy, the management does not manipulate the financial statements to the point of signalling (falsely) a good financial situation. The good financial condition seems truly and fairly represented in financial statements, despite the subsequent bankruptcy of our sample companies.

The results contradict conclusions of several recent research on the relation of various earnings quality measures with bankruptcy prediction. In general, earnings management is more typical for failing companies. The closer to the moment of bankruptcy of the company, the lower the quality of earnings [2]. Higher chances of financial distress are also positively correlated with higher utilization of earnings management [6], although contrary evidence was also provided [9]. Much of the previous research also supports the correlation between lower earnings quality and higher probability of bankruptcy [3].

However, we should emphasize the important difference between those studies and ours: we used a different measure of the quality of financial reporting. The earnings quality used in those studies reflect much, but not all of the financial reporting quality. We used metrics based on Benford’s law, which substitute for any manipulations and errors in financial statements items [11]. Amiram et al. [10] proved that conformity with Benford’s law is significantly correlated with other measures of financial reporting quality: Financial statements that demonstrate greater conformity with Benford’s law also have higher quality of earnings. Therefore, we might expect that financial statements of failed companies complied less with Benford’s law and that signalling financial distress could have motivate their managers to distort accounting numbers. Finally, we proved that such manipulation existed; however, the manipulation does not cause financial statements to look good enough to distort bankruptcy prediction model. In other words, managers of failing companies might manipulate their financial statements, but not to the point of turning a bankrupt company into prosperous-looking.

## Conclusions

The results of the study do not support the hypothesis. Accounting manipulations or other deficiencies in the quality of data in the financial statements are nor the reason for the unreliability of the Altman model. An inverse relationship was demonstrated: The higher the probability of bankruptcy, as measured by Altman’s Z-score, the lower the quality of the accounting data. This result seems to confirm the interpretation of the Z-score as a measure of the financial health of a company. The worse the financial health of an entity, the greater the likelihood of accounting frauds. Therefore, companies characterized by poorer financial health publish financial statements of poorer quality. This effect was confirmed in our study on bankrupt companies. The correlation of deviations from Benford’s law and other measures of financial health needs to be tested on a wider research sample.

Our results reveal that a deterioration in the quality of financial statements may be a signal of an increasing probability of bankruptcy. It is possible to improve the predictive properties of bankruptcy prediction models by including variables that quantify the probability of manipulation in their design. As expected, the accuracy of the indications of bankruptcy prediction models increases as the moment of bankruptcy approaches. In contrast, there is no clear evidence of the impact of the importance of company size.

Therefore, companies in distress cheat, but are not able to influence the results of the Altman model. In other words, the Altman model is to some extent robust to manipulation because the sample, on which it was built could also contain manipulated data. Therefore, the unreliability of the Altman model is due to reasons that are not predictable from the financial statements.

The main limitation of our study is our sample. We tested the relationship with failed Polish private companies. Further studies might investigate the reasons for the failures of other bankruptcy prediction models in a larger data set. The other direction of further studies is to test the relation between financial condition (measured with Altman’s model or with other metrics) and accounting data quality measured as on the basis of Benford’s law.

## Supporting information

S1 File Dataset for models estimation. (CSV)
